# Plasmonic Light Trapping in Thin-Film Solar Cells: Impact of Modeling on Performance Prediction

**DOI:** 10.3390/ma8063648

**Published:** 2015-06-18

**Authors:** Alberto Micco, Marco Pisco, Armando Ricciardi, Lucia V. Mercaldo, Iurie Usatii, Vera La Ferrara, Paola Delli Veneri, Antonello Cutolo, Andrea Cusano

**Affiliations:** 1 Division of Optoelectronics, Engineering Department, University of Sannio, Benevento 82100, Italy; E-Mails: alberto.micco@unisannio.it (A.M.); pisco@unisannio.it (M.P.); aricciardi@unisannio.it (A.R.); cutolo@unisannio.it (A.C.); 2 ENEA–Portici Research Center, Portici 80055, Italy; E-Mails: lucia.mercaldo@enea.it (L.V.M.); iurie.usatii@enea.it (I.U.); vera.laferrara@enea.it (V.L.F.); paola.delliveneri@enea.it (P.D.V.)

**Keywords:** solar cells, plasmonics, light trapping

## Abstract

We present a comparative study on numerical models used to predict the absorption enhancement in thin-film solar cells due to the presence of structured back-reflectors exciting, at specific wavelengths, hybrid plasmonic-photonic resonances. To evaluate the effectiveness of the analyzed models, they have been applied in a case study: starting from a U-shaped textured glass thin-film, µc-Si:H solar cells have been successfully fabricated. The fabricated cells, with different intrinsic layer thicknesses, have been morphologically, optically and electrically characterized. The experimental results have been successively compared with the numerical predictions. We have found that, in contrast to basic models based on the underlying schematics of the cell, numerical models taking into account the real morphology of the fabricated device, are able to effectively predict the cells performances in terms of both optical absorption and short-circuit current values.

## 1. Introduction

Nanophotonics and plasmonics are spurring a wide variety of new research aimed at improving the performance of thin-film solar cells [1,2] limited by the finite size of the active region. Advanced photon management strategies and light trapping schemes have gained tremendous interest as a result of their abilities to make the cell “optically thick” by scattering incident light and elongating the optical path inside the cells, thus improving the cells’ efficiencies [3,4,5]. 

In particular, backreflectors, such as metallic diffraction gratings, can couple incident light at specific wavelengths into resonant (photonic) modes propagating along the plane of the cell [6] and into plasmonic polaritons excited at the metal/semiconductor interface [1,7,8].

Backreflectors with different geometrical features [9,10,11,12,13], gratings symmetries [14,15], and periodic and aperiodic configurations [16,17,18,19,20] have been reported. 

To maximize the trapping of light and thus enhance the photocurrent across the entire solar spectral range, an appropriate design of the metallic backreflector is of the utmost importance. In this regard, numerical modeling is an extremely useful tool for correctly predicting the cell performances, thereby providing time and cost savings.

Many numerical studies have been proposed, most of them demonstrating that the real morphology of the cell has to be considered in the numerical models to correctly predict the cell performances in terms of light absorption [15,18,21,22,23,24]. Specifically, Ferry *et al.* performed a study on cell modeling and optimization using experimental cross-sections and morphological data as a guide to simulate realistic cell architectures [22]. Similarly, Čampa *et al*. also optimized a 1-D backreflector using a numerical model that considers the morphologies of the cells retrieved by atomic force microscopic analysis. Steltenpool *et al.* studied the influence of the texture shape to optimize micro-morph cell performances using a realistic, periodic, V-shaped texturing [24]. 

However, even if avoiding irrelevant details is a general rule in numerical modeling toward saving computational resources, to what extent the correct texturing model affects the numerical predictions of the performances of thin-film solar cells remains to be investigated. Specifically, it is worth investigating the influence of the morphological changes, occurring during cell growth, on the light trapping capability of backreflectors via photonic and plasmonic resonance excitations.

The effect of the real morphology on the numerical predictions of the cell performances can be less or more significant depending on the fabrication processes exploited. For a bottom-up method, the fabrication process starts from the definition of the backreflector, and thus, its characteristics are already defined at time zero, correctly resembling the design parameters. 

However, when a top-down approach is used (when the cell is built starting from a textured glass), the geometrical properties of the metallic backreflector are strongly influenced by the different layer depositions that inevitably modify the initial shape of the textured glass. As a result, depending on the number of deposited layers as well as their thickness, the morphology of the backreflector becomes significantly different than that of the textured substrate, whose parameters have been optimized in the design phase. In addition, the geometrical characteristics of the textured glass play a crucial role in determining the final morphology of the backreflector. Typically, the conformal deposition of the layers composing the cell tends to smooth steep textures such as pyramidal structures and 2D gratings with perpendicular holes. Moreover, the aspect ratio of the textured surface is not retained by the backreflector.

Clearly, all of these effects have to be considered in a model that attempts to effectively study the absorption enhancement due to the presence of a metallic backreflector. Moreover, the phase matching condition at the basis of the excitation of both photonic and plasmonic modes strongly depends on both physical and geometrical characteristics of the structures involved into the coupling process. For example, discrepancies in terms of the filling factor or thickness of the backreflector may result in a shift of the resonant wavelengths, with a consequent modification of the absorption spectrum, thus generating a disagreement between experimental measurements and numerical predictions.

Based on these considerations, in this work, we propose a comparative study of numerical models used to predict the absorption enhancement in thin-film solar cells with structured back reflectors fabricated with a top-down approach. For our study, we decided to use a textured glass as a reference, which has been already proven to guarantee very good light trapping performance [24]. Thin-film μcSi:H solar cells have thus been fabricated as test cells. A careful morphological analysis has been conducted before and after cell growth to retrieve the geometrical parameters used in the numerical model. 

The rest of the paper is organized as follows. First, we present the experimental part, which contains a brief description of the fabrication process and the results of both optical and electrical measurements. Then, we report a detailed morphological characterization of the fabricated samples outlining the dependence of the pattern transferring on the intrinsic layer thickness while the second part addresses the description of the numerical models used, including an analysis of the resonant phenomenon responsible for the absorption enhancement. Finally, the numerical results are compared with the experimental data to evaluate the effectiveness of the proposed model.

## 2. Fabrication Process and Electrical Performances of the Cells

**Figure 1:**
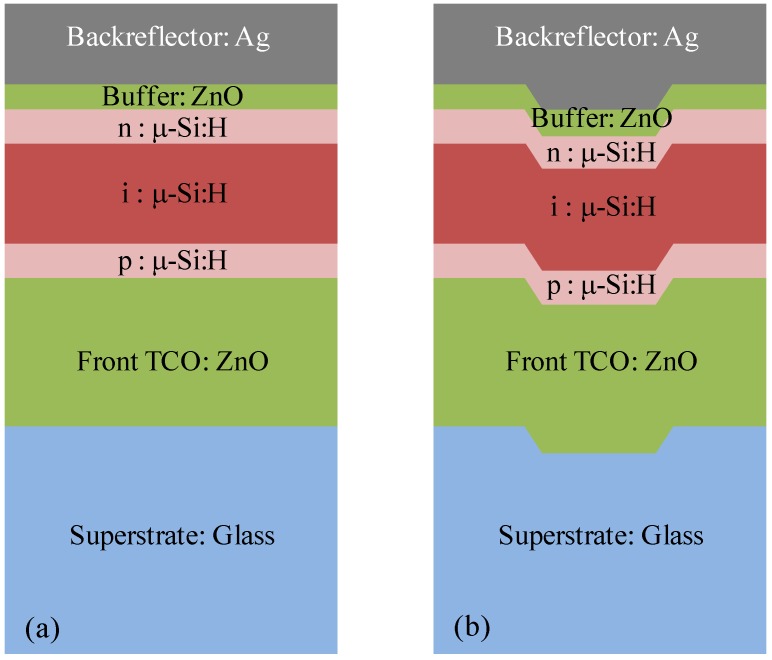
Schematic of the layers that constitute the fabricated solar cells for flat (**a**) and textured (**b**) substrates.

Superstrate-type thin-film µc-Si:H solar cells are schematically depicted in Figure 1a. The cell essentially consists of a glass superstrate, a thick layer of doped ZnO, that is a transparent conductive oxide (TCO) acting as front electrode, a μc-Si:H p-i-n junction for the optoelectronic conversion, a thin doped ZnO layer acting as a buffer, and a final Ag layer used as backreflector and back contact.

In this configuration, the solar cells are grown following a top-down fabrication process, *i.e.*, starting with the deposition onto glass of the TCO layer, and finishing with the deposition of the metal backreflecting contact after the deposition of the silicon p-i-n junction. In this approach, the texture grating is realized in the starting glass superstrate, and the pattern is automatically transferred to all the interfaces with features depending on the thickness of the involved layers.

To analyze the effect of the growth of the cells on the texture, we have realized two sets of solar cells, with different thicknesses of the active µc-Si:H layer. Additionally, we also fabricated cells on flat glass to be used as baseline.

To obtain a meaningful comparison, the µc-Si:H p-i-n solar cells were co-deposited on flat and textured glass superstrates to minimize the variations in the thicknesses of the layers due to the fabrication tolerances of the deposition process.

Glass with a 2D nano-imprinted grating obtained from OM&T Moser Baer Technologies (now Morphotonics, Veldhoven, The Netherlands) was used as the textured superstrate [24]. The texture is composed of a rectangular lattice of holes with a period of 600 nm and a depth of approximately 100 nm. Figure 1b schematically represents the cell layer fabricated on the textured glass.

The nominal thicknesses chosen for the layers are the following: 800 nm for the front TCO, 800 nm and 1500 nm for the silicon p-i-n stack, 80 nm for the ZnO buffer layer and 200 nm for the Ag. In the p-i-n junction, the *p*-layer and the *n*-layer are 30 nm thick.

**Figure 2:**
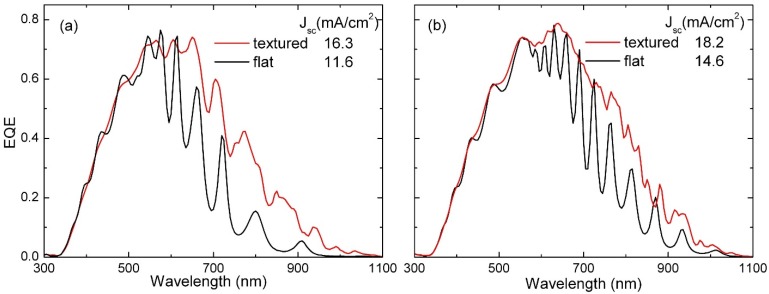
External quantum efficiency of identical µc-Si:H solar cells co-deposited on nanotextured (red line) and flat (black line) glass substrates, with absorber layer thicknesses of 800 nm (**a**) and 1500 nm (**b**). The short-circuit current density reported in the legend has been extracted from the EQE curve, as explained in the Materials and Methods section.

The flat and textured p-i-n µc-Si:H solar cells were characterized by measuring the current density-voltage J(V) characteristic under AM1.5 illumination using a solar simulator and the external quantum efficiency (EQE). From the J(V) curves, similar electrical performances were observed for all of the devices. The open-circuit voltage V_OC_ and fill factor FF are ~500 mV and 65%, respectively. As expected, different short-circuit currents are measured as an effect of absorber thickness and use of flat or textured interfaces. The contribution at different wavelengths to the generated current for all of the cells is shown in Figure 2 by means of EQE spectra, where the flat (black lines) and textured (red lines) co-deposited cells are compared for the two absorber thicknesses. This measurement allows evaluating the real optical improvement achievable using the nanotexture, including charge collection effects. In both the cases, the EQE of the cell grown on the nanopatterned substrate is higher than the flat baseline cell demonstrating the effectiveness of the structured backreflectors used in this study. In particular, the significant EQE improvement in the long wavelength range (above 600 nm) is indicative of a relevant increase in the light path as a result of multiple passes of scattered light within the solar cell. In addition to light scattering and diffraction at the textured interfaces, photonic and plasmonic resonances may simultaneously contribute to the light trapping process. Moreover, a reduction in the interference fringes is observed in the spectra due to the partial loss of coherence of the scattered light at the textured interfaces, possibly accompanied by a slight decrease in the reflection losses. Independently of the physical mechanisms at play, the significant EQE enhancement corresponds to a relevant increase in the photocurrent with respect to the flat substrate. The short-circuit current density values (*J*_SC_), directly evaluated using the EQE curves, are reported in the legend of Figure 2. Quantitatively, when switching from the flat to the textured glass, the increase (Δ*J*_SC_) is 3.6 and 4.7 mA for the thicker and thinner cell, respectively. The larger enhancement observed for the 800-nm-thick device might be indicative of a more relevant effect from the texturing in the case of reduced thickness, although there could also be some influence from the charge collection efficiency that is typically higher for reduced charge paths.

## 3. Morphological and Spectral Characterizations

### 3.1. Morphological Analysis of the Cells

A morphological analysis was conducted on the pristine glass superstrate and at the bottom of the fabricated cells to study the variations in the texture shape occurring during the growth of the cells. 

As mentioned above, the texture on the glass superstrate is characterized by a square lattice of holes with nominal period of 600 nm and depth of approximately 100 nm. 

**Figure 3:**
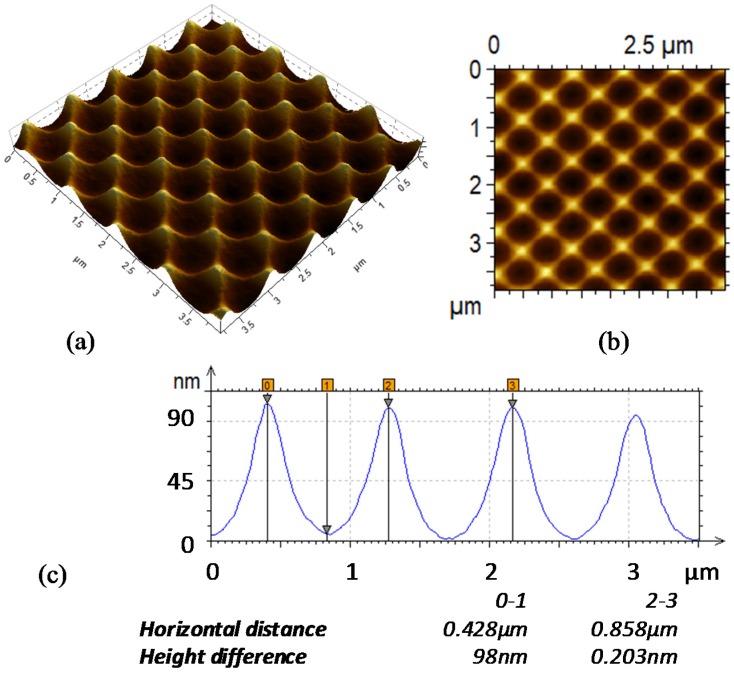
Atomic force microscopy topography of the textured glass superstrate: (**a**) 3D topographic image; (**b**) 2D topographic image; (**c**) cross-section profile in the diagonal direction with respect to the lattice.

In Figure 3, the Atomic Force Microscopy (AFM) topographic image of the texture of the glass superstrate is reported. The texture has a particular shape featuring a regular pattern of U-shaped valleys. From the top view of the topography image of the superstrate glass, reported in Figure 3b, we can observe holes with a circular shape and ridges similar to four-pointed stars. This particular shape has been selected as a trade-off between a high aspect ratio texture, which is useful for achieving the best light trapping performance, and an open texture, which ensures optimum electrical performance [24]. It is well known that excessively steep textures, such as binary surface-relief gratings and pyramidal textures with V-shaped valleys, often induce defects in μc-Si:H films, causing cell shunting and impairing the photovoltaic performances [25].

After the deposition of the front TCO and Si stack constituting the solar cells, we conducted another morphologic characterization. 

**Figure 4:**
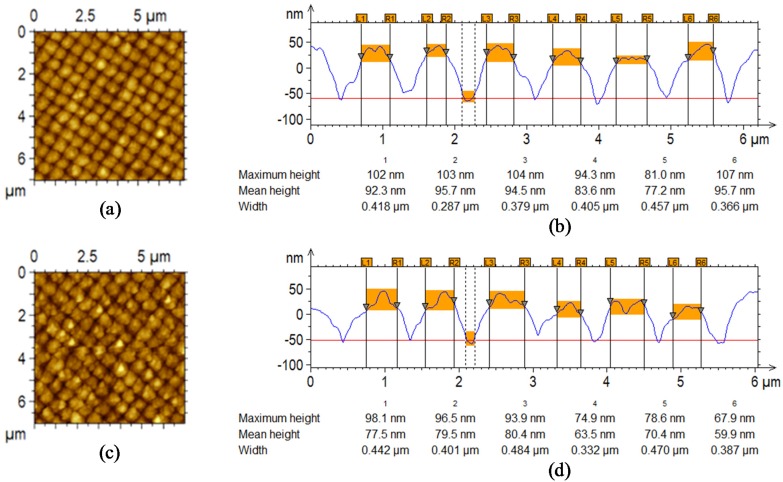
AFM topography images of the fabricated solar cells with cross section profile: (**a**–**b**) for the cell with 800-nm-thick Si stack; (**c**–**d**) for the cell with 1500-nm-thick Si stack.

In Figure 4, the morphological AFM images and the cross-section profiles acquired at the back Si surface of the cells with Si thickness of 800 nm and 1500 nm are displayed.

As observed in Figure 4a,c, the bottom surface still presents a periodic pattern despite being less defined and regular. Nevertheless, by analyzing the texture before and after deposition, further differences can be easily appreciated. Observing the same image after the deposition process of the cells (Figure 4c,d), the large black holes of the glass superstrate disappeared and, in place of the small ridges, small holes are visible. Basically, from the morphological viewpoint, the ridges are replaced by circular shapes, and the holes become square ridges. This effect can be explained by the conformal deposition typical of the Plasma Enhanced Chemical Vapor Deposition (PECVD) process [26]: the growth of the materials gradually modifies the original grating morphology in such a way that the holes tend to decrease in diameter because of the thickening of the hole sides. 

**Figure 5:**
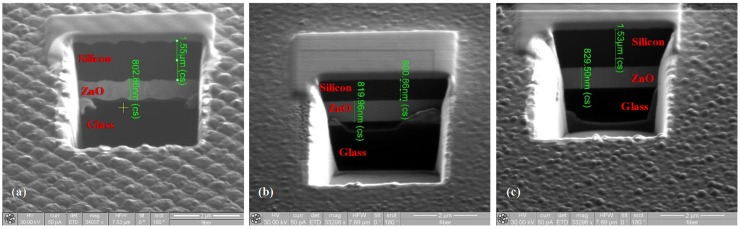
Cross-section of the fabricated solar cells for the textured cell (**a**) and for the cell fabricated on flat substrates with silicon thicknesses of 800 nm (**b**) and 1500 nm (**c**). From the measurements, the thicknesses for the ZnO layer are approximately 820 nm and 830 nm, and those for the silicon layer are 800 nm and 1530 nm for the cell with a nominal silicon layer thickness of 800 nm and 1500 nm, respectively.

The changes in the morphology are confirmed by the cross-section reported in Figure 5a, for the thicker cell obtained using a Focused Ion Beam (FIB) milling machine. In the image, all of the layers of the cell are distinguishable; moreover, changes occurring in the texture profile after silicon deposition are clearly visible.

Based on these observations, we performed a quantitative analysis of the morphological features highlighted by the AFM image (Figure 3c and Figure 4b–d) to obtain a growth rule for the grating parameters, in particular for the height and the width of the holes, at various cell thicknesses. Specifically, we measured the hole depth and width using the analysis software of the AFM.

By comparing the cross-profiles, which are reported in Figure 3c and Figure 4b–d, related to the superstrate textured glass and the two fabricated cells, respectively, we quantified the variation in the height of the grating and the shrinkage of the holes. 

**Figure 6:**
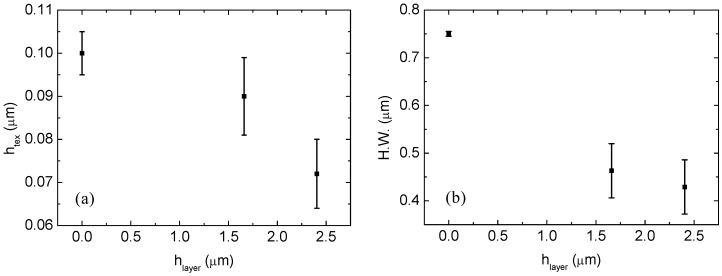
Experimental data for the grating height (**a**) and hole width parameters (**b**) obtained from the AFM image cross-sections.

In Figure 6a, the measured depths of the grating are plotted as a function of the cell thickness (front electrode plus p-i-n Si stack) for which the AFM inspection is performed, where the zero value is set at the textured substrate. In the same graph, we also report the error bars around the medium values that are due to the deposition process tolerances. After the cell growth, a meaningful reduction in the height of the grating can be observed from the original size of 100 nm down to 90 ± 8 nm and 72 ± 9 nm for the complete cell with 800-nm- and 1500-nm-thick silicon layers, respectively.

Similarly in Figure 6b, we show the hole widths *versus* the thickness. We observed a reduction in the hole diameters from approximately 750 nm down to 463 ± 57 nm and 429 ± 57 nm for the cells with 800-nm- and 1500-nm-thick silicon layers, respectively.

The FIB cross-sections of the cells deposited on the flat substrates are shown in Figure 5b,c. Thicknesses of approximately 820 nm and 830 nm (for the ZnO front contact) and approximately 800 nm and 1530 nm (for the silicon p-i-n region) have been measured for cells with nominal silicon thickness of 800 nm and 1500 nm, respectively. The small deviations between nominal and measured values are within fabrication tolerances.

The texturing feature sizes of the fabricated cells are used in the numerical analysis in the following section to estimate the geometrical grating features also at intermediate interfaces within the cells. The information retrieved from the FIB cross-sections are also used in a numerical investigation to refine the thickness values of the ZnO and p-i-n layers.

### 3.2. Optical Characterization of the Cells

We also performed a spectral characterization of the fabricated cells. Specifically, we retrieved the optical reflectance of the cells using a simple setup exploiting a reflection probe connected to a light source and a spectrophotometer (additional details can be found in the methods section). 

The spectral measurements are conducted to provide as consistent of a comparison with the numerical results as possible. The EQE measurement is affected by not only the optical characteristic of the materials but also the electrical properties of the p-i-n junction. Thus, in order to compare the numerical and experimental results, we used a pure optical observable to neglect the charge collection effects, which are not considered in the numerical model. The reflection measurements are performed with normal light incidence, optically resembling the ideal numerical scenario.

**Figure 7:**
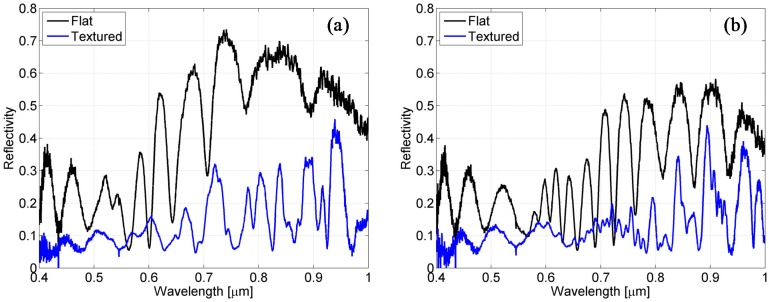
Optical characterization of the solar cells, with reflectance spectra for the flat superstrate (solid black line) and textured superstrate (solid blue line) with Si layer thickness of 800 nm (**a**) and 1500 nm (**b**).

In Figure 7a,b, the measured reflectivity spectra for the cells with nominal Si layer thickness of 800 nm and 1500 nm, respectively, are shown. In the same graphs, the reflectance of the co-deposited counterpart with the flat superstrate is shown.

The reflection spectra of the flat cells resemble the reflection spectra of a multilayer Fabry-Perot interferometer, featuring interference fringes with a slowly varying envelope. The textured cells instead produce a low reflectance baseline with reflectance peaks, which are not easily attributable to interference fringes. 

Furthermore, from the comparison, the overall reflectivity reduction of the cell with textured glass (solid blue lines) compared to the cell with flat superstrate (solid black lines) is evident for both thicknesses. 

This reflectance reduction can be attributed to the strong light scattering and diffraction occurring at the textured interfaces but can also be due to photonic and plasmonic resonances that contribute to the light trapping process. The following numerical section will ascertain the role played by such resonances in the cell spectral features.

## 4. Numerical Analysis and Experimental Validation

Two numerical models are implemented to evaluate the performances of the solar cells. All of the numerical simulations are performed using the finite element method. Both the flat and textured cells are modeled with a unit cell with periodic boundary conditions (additional details on the numerical simulation methodology can be found in the methods section). The reflectance of the simulated cell is calculated using the scattering parameters. The cell absorbance is obtained using the reflectance by supposing no transmission through the metallic back contact. 

**Figure 8:**
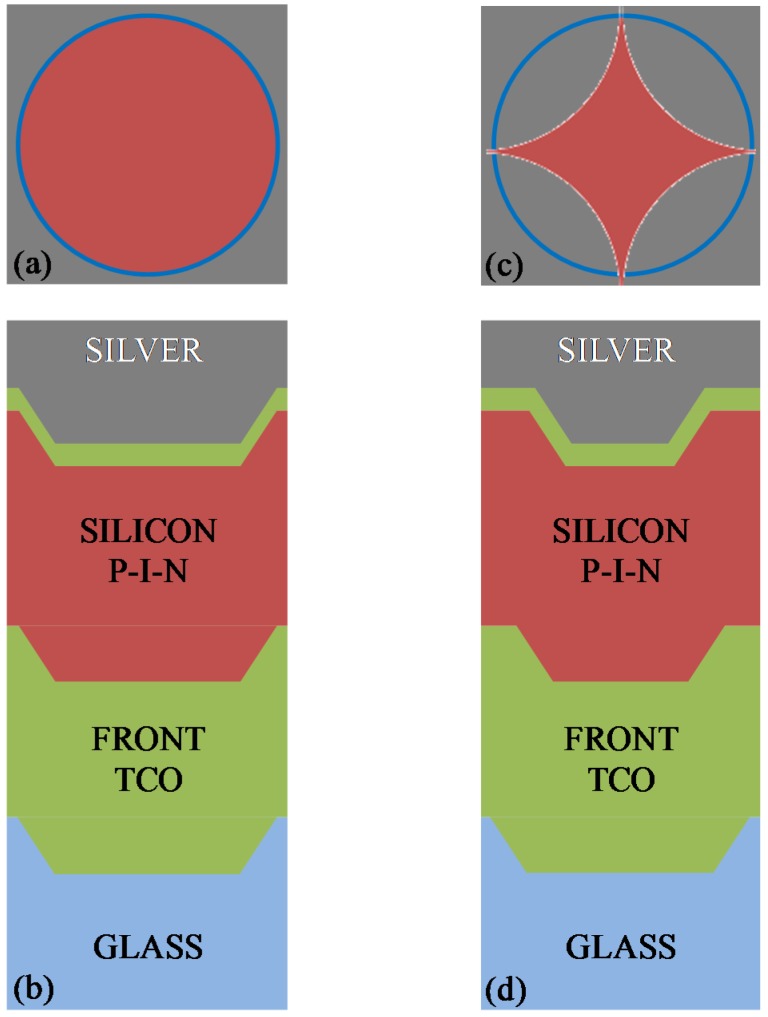
Schematics of the implemented numerical models: (**a**) top view and (**b**) cross-section of the first model, where the deposition effects are neglected; (**c**) top view and (**d**) cross-section of the second model, where the growth effects are considered as observed by AFM measurements.

In the first model, whose schematic is shown in Figure 8a,b, the holes are modeled by a truncated cone shape, resembling the shape of the texture superstrate. As shown in the cross-section in Figure 8a, the texture shape is modeled in the same way in each layer of the structure, neglecting the effects of materials growth. Consequently, from the top view in Figure 8b, we observe that the holes of the superstrate (in blue) and those at the back side of the cell (in brown) are all aligned and circularly shaped.

Figure 8c,d show the schematic of the second model, where the holes are again modeled as truncated cones, but the conformal deposition effects are considered by exploiting the results of the morphological characterization. Essentially, we consider in the model the meaningful reduction in size of the holes observed during cell growth. Although the original holes in the superstrate are circular, those observed after the growth of the entire cell exhibit a four-pointed star shape.

In both of the models, the p-i-n junction is represented as a single silicon layer because the intrinsic layer is very thick compared to the p and n regions.

For the thicknesses of the various layers, we refer to the nominal values set during the controlled deposition process and those measured with FIB cross-sectioning reported in the previous section. The refractive indexes of materials used in the analysis are retrieved from experimental ellipsometric measurements (see the Materials section for details).

**Figure 9:**
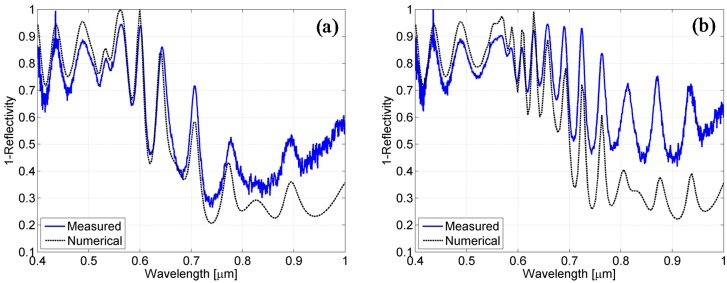
Comparison between simulated (dotted black line) and measured (solid blue line) absorption spectra for the cells grown on flat glass with nominal Si thickness of 800 nm (**a**) and 1500 nm (**b**). From the numerical analysis, a thickness of 800 nm is obtained for the ZnO layer and 780 nm and 1525 nm for the silicon layers.

As a first step in our numerical analysis, we have focused our attention on the numerical agreement with the experimental results for the flat cells. To match the absorbance data measured on the solar cells fabricated on flat superstrates, we performed parametric simulations by considering the results of our morphological analysis. Specifically, we conducted a parameterization in the range of ±50 nm (*i.e.*, tolerance ranges of the deposition process) and in steps of 5 nm around the measured ZnO and Silicon thickness. The resulting numerical absorbance spectra are reported in Figure 9a,b for the optimal values.

A good agreement has been obtained for a ZnO thickness of 800 nm and silicon thicknesses of 780 nm and 1525 nm (in place of the nominal values of 800 nm and 1500 nm, respectively). The interference fringes (in terms of period and spectral position) are in good agreement, thus demonstrating a correct estimation of the optical path even if differences in the absorbance amplitude can still be observed, especially for wavelengths exceeding 600 nm. This mismatch, albeit acceptable, can be attributed to a slight underestimation of the materials dispersive losses or to the lateral out-coupled light from the marginal areas of cells [27]. Despite these numerical differences, overall, the short-circuit current remains in good agreement, with an estimated value of 12.41 mA/cm^2^ for the cell with a 800 nm thickness *versus* 11.6 mA/cm^2^ measured and an estimated value of 14.77 mA/cm^2^*versus* 14.6 mA/cm^2^ measured for the cell with a 1500 nm thickness. 

In the following simulations, the thicknesses obtained with the flat cells are also used for the co-deposited cells grown on textured superstrates.

**Figure 10:**
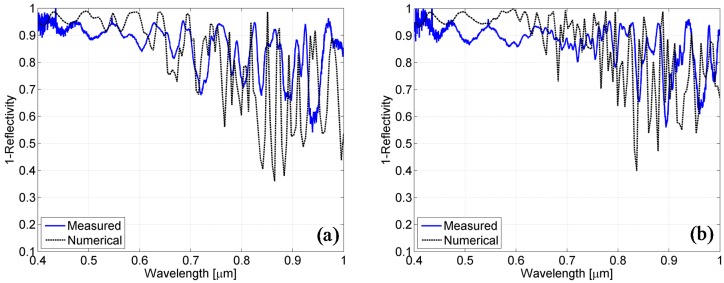
Comparison between the spectra obtained from the simulation using the first model (dotted black line) and those measured on the cells (solid blue line) for the cell grown on the textured superstrate with a nominal thickness of silicon of 800 nm (**a**) and for that of 1500 nm (**b**).

In Figure 10, the measured absorbance spectra for the textured cells (solid blue line) are compared with those retrieved from the numerical simulations (dashed black line) using the simplified model. Specifically, Figure 10a,b show the absorbance for the cells with nominal thickness of silicon of 800 nm (a) and of 1500 nm (b). As evident, all of the spectra feature a high level of absorbance and several absorptive peaks. Despite a general agreement on the absorbance levels, the numerical and experimental absorbance spectra cannot be reasonably correlated in terms of spectral resonances. 

**Figure 11:**
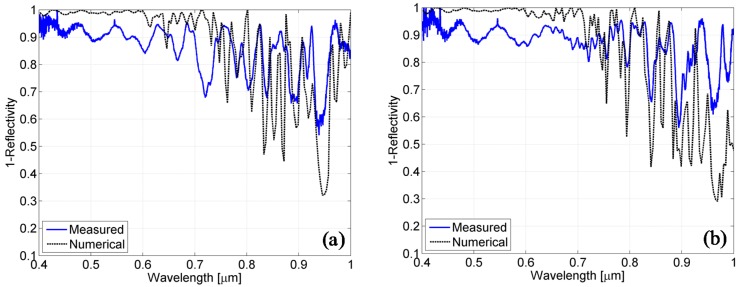
Comparison between the spectra obtained from the simulation of the second model (dotted black line) and those measured on the cells (solid blue line) for the cell growth on the textured superstrate with a nominal thickness of silicon of 800 nm (**a**) and for that of 1500 nm (**b**).

In Figure 11, the measured absorbance spectra (solid blue line) are compared with those retrieved from the numerical simulations (dashed black line) using the model taking into account the growth effects on the texture. In particular, even if differences in the peak amplitudes can still be observed, a good correspondence among the spectral positions of the theoretical peaks and those effectively measured into the structures can be clearly observed. 

**Figure 12:**
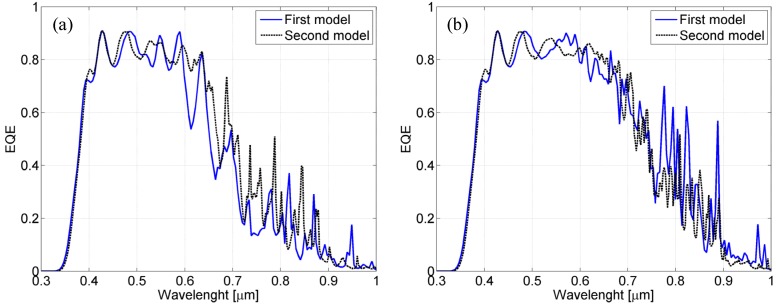
EQE spectra retrieved from the simulation with the simplified and improved numerical models for the textured cell with 800-nm-thick (**a**) and 1500-nm-thick (**b**) silicon layers.

A comparison using a global performance parameter such as the short-circuit current may be more effective. Therefore, we calculate the absorption over the photocurrent-generating region by integrating the resistive heating losses over the µ-Si:H region. In Figure 12, we show the resulting numerical absorption spectra of the cells, which are calculated with both models. 

By comparing these spectra with the experimental ones in Figure 2, a general agreement can still be observed. In particular, we notice that the absorbance is very low, below 400 nm, and that in the range 400–600 nm, the absorption spectra are characterized by oscillations (typical of the Fabry-Perot effect). In contrast, in the long wavelength range (above 600 nm), a significant absorbance improvement is evident, and the Fabry-Perot envelope is interrupted by several absorption peaks. 

Note that this absorption spectrum coincides with the EQE if we assume that all of the absorbed power produces carrier excitations (hypothesis of perfect carrier collection). Further integrating this spectrum over wavelength yields the short-circuit current density *J*_SC_. 

Numerical values of the short-circuit currents have been calculated for both models and compared with the values obtained from the experimental characterization. Obviously, the larger silicon layer thickness, the larger is expected to be the *J*_SC_ values. As a consequence, the ratio between *J*_SC_ of textured cell with 1500-nm-thick and 800-nm-thicksilicon layer, has been evaluated. This current enhancement is 11.6% in the experimental case. The same quantity has been evaluated also by using the two different numerical models discussed above. Also in this case, only the result achieved with the improved numerical model is in good agreement with the measured values. In fact, the simplified model provides a significantly different current enhancement of 27.4%, more than twice the measured value. On the other hand, the same quantity evaluated with the improved model is 11.53% that matches quite well the experimental data.

Overall, the comparison between the numerical and experimental data clearly demonstrates that a correct modeling of textured cells needs to properly consider the conformal geometry and optical properties of each material. Nevertheless, the growth effects, occurring during cell fabrication, also have to be carefully considered because they lead to a significant variation in the feature sizes in the successive conformal layers that is detrimental to the accuracy of the numerical prediction of performance.

### Photonic and Plasmonic Contribution to the Light Trapping

The predictions obtained from the two models mainly differ in terms of number and positions of the spectral resonances. In the present section, we attempt to determine the nature of these peaks by exploiting the physical insight capability supplied by the numerical analysis to better understand the factors affecting the spectral positions of the absorbance peaks.

To determine the nature of the absorption peaks, shown in Figure 12 on the red side of the solar spectrum, we carefully examined the electric field distributions and the associated losses in the metallic and dielectric domains of the textured cells. Furthermore, to understand the role played by photonic/plasmonic resonances in terms of the efficiency of the light trapping process, we separated absorption in parasitic materials (silver and TCO) from absorption in photocurrent generating materials (µc-Si:H), even over irregularly shaped features.

**Figure 13:**
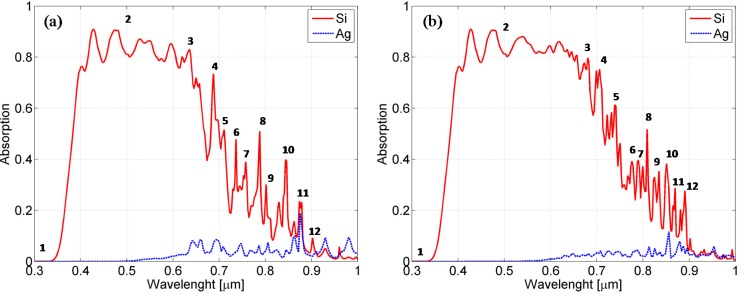
Absorption spectra evaluated over the silicon (solid lines) and into the silver (dotted lines) for the cells with silicon layer thicknesses of 800 nm (**a**) and 1500 nm (**b**).

To this aim, in Figure 13, the absorption spectra calculated over the silicon and silver region are displayed and compared to the entire considered wavelength range for both textured cells.

The absorption associated with the silicon region is obviously responsible for the current generation. In our analysis, we found that the absorption over the silicon mainly follows the spectral behavior of the EQE, as shown in Figure 13. At wavelengths smaller than 400 nm, the absorption is low because the ZnO is not transparent. The absorption becomes high as soon as the ZnO allows the transmission of light and starts decreasing at wavelengths greater than 600 nm, where it also presents several sharp absorption peaks for wavelengths exceeding 600 nm.

In Figure 13, we also show the resistive heating losses over the silver. We calculated the integral of the time-averaged resistive heating over the silver because these losses, even if not useful for increasing the cell performances, are a useful indicator of the excitation of surface plasmons [28]. They are absent up to 500 nm, whereupon they slightly increase, and several peaks at specific wavelengths can be observed in the 600–1000 nm range. 

**Figure 14:**
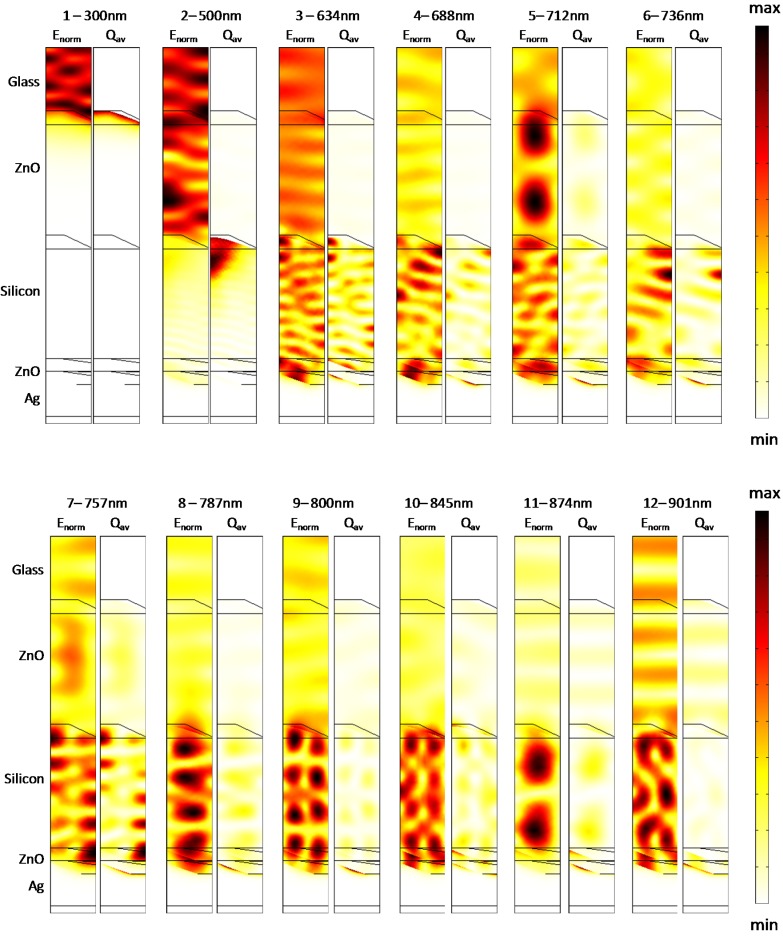
Normalized electric field and resistive heating field distributions along an orthogonal slice in the *zx* plane for the cells with an 800-nm-thick silicon layer at individual wavelengths.

**Figure 15:**
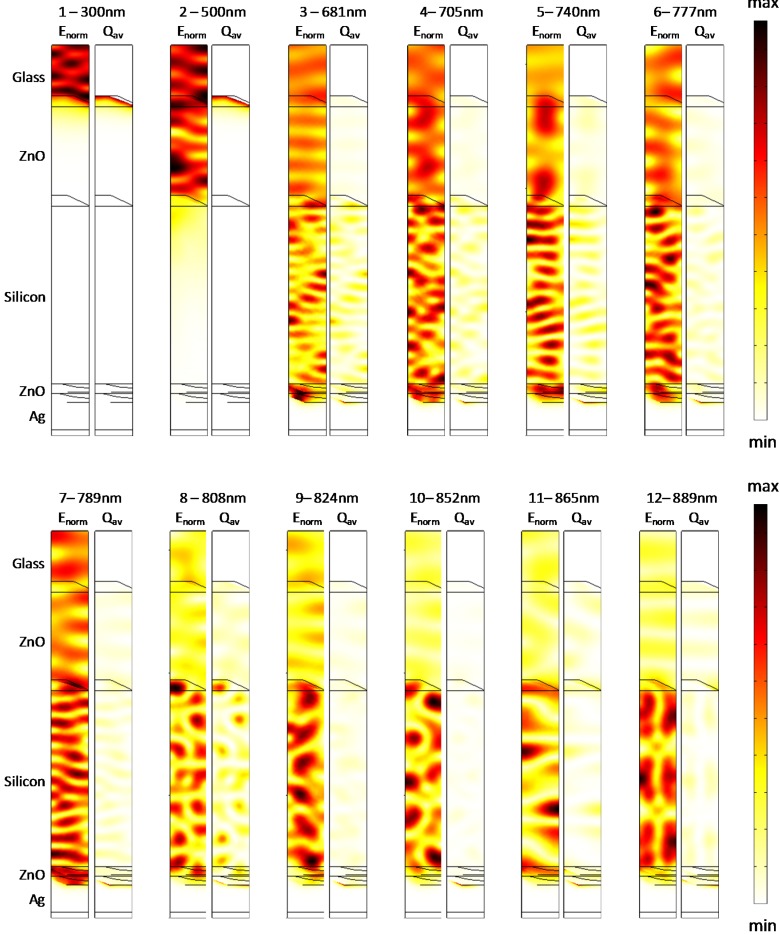
Normalized electric field and the resistive heating field distributions along an orthogonal slice in the *zx* plane for the cells with a 1500-nm-thick silicon layer at individual wavelengths.

We examine the electromagnetic field distributions within the active region to obtain insights into the mechanisms leading to enhanced absorption. In Figure 14, we show the normalized electric field (*E*_norm_) and the resistive heating field (*Q*_av_) distributions along an orthogonal slice in the *zx* plane (centered in the computational domain) for the textured cell with a silicon thickness of 800 nm. Specifically, the field distributions are shown at the wavelengths marked in Figure 13a when the absorption sharply increases to capture the light interaction mechanism with the patterned backreflectors. Figure 15 shows the same data as in Figure 14, but here, the cell features a 1500-nm-thick µc-Si:H region, and the wavelengths were similarly selected from the absorption spectrum in Figure 13b. Minor peaks have been neglected for the sake of simplicity. Additionally, we also show the field map distributions at individual wavelengths, namely, 300 nm and 500 nm, to elucidate the mechanisms that contribute to the absorption in the other regions of the solar spectrum.

As evident at 300 nm, the absorption is confined into the top portion of the cell, in agreement with the fact that the ZnO is not transparent up to approximately 400 nm. Consequently, in this wavelength range, the incident light does not interact with the metal back contact. At the intermediate wavelength of 500 nm, a standing wave pattern can be observed as a result of the predominant Fabry-Perot interaction. At higher wavelengths, when they approach the texturing size, light is scattered by the metal backreflector, as highlighted by the increasing losses occurring in the silver region. The growing involvement of the silver region in the parasitic losses is slightly detrimental but is also an evidence that the metal nanostructures at the backreflector are providing scattering into photonic modes, thereby leading to increasing absorption in the photocurrent generating region (Figure 13). Note that the contribution of the plasmonic resonances to the carrier collection cannot be evaluated in a straightforward manner in a single simulation; however, it is reasonably negligible because it is only limited to the electromagnetic field tails, able to reach the silicon region, by overpassing the 80 nm ZnO buffer layer. 

The light coupling to waveguide modes can instead be clearly appreciated by observing the intensity profile maps due to the scattering from the periodic pattern. Here, the absorption in the silicon region is very strong, and the regular periodic profiles can be attributed to the excitation of waveguide modes. The absorption peaks wavelengths are dictated by the phase matching condition and are in turn related to the mode field and thus to the geometry of the cell. Nevertheless, from the field distributions, it can be appreciated that the incident light interacts with metal back contact as well as with the intermediate scatterers, particularly with those composed of the conformal interface between the front TCO and the µc-Si:H. 

From this viewpoint, it is easily understandable that the correct estimation of the feature sizes in the intermediate layers during cell growth strongly affects the spectral absorption peaks and is responsible for a correct numerical prediction via the excitation of the guided modes in the cell. These modes are in turn mainly responsible for the narrowband absorption enhancements and consequently for the differences emerging in the two investigated models. 

## 5. Materials and Methods

### 5.1. Deposition Process of the Cells

For a meaningful comparison, the µc-Si:H p-i-n solar cells were co-deposited on flat and textured glass superstrates. Glass with a 2D nano-imprinted grating by OM&T Moser Baer Technologies (now Morphotonics) was used as the textured superstrate. As the front transparent electrode, an 800-nm-thick Al-doped ZnO layer was deposited by RF magnetron sputtering. The ZnO:Al films, conformally grown on the textured glass substrates and on the flat witness glass plates, are characterized by high optical transmission (approximately 80%) and low sheet resistance (~10 Ω/sq). The p-i-n silicon stack was subsequently deposited using a commercial cluster tool system (MVSystems Inc., Golden, CO, USA) equipped with standard and very-high-frequency (VHF) plasma-enhanced chemical vapor deposition (PECVD) reactors. A mixture of silane (SiH_4_) and hydrogen (H_2_) was used as the source gas, with an additional dopant gas for the doped layers, namely, trimethylboron (TMB) and phosphine (PH_3_) for p-type and n-type doping, respectively. The structure consisted of an intrinsic µc-Si:H layer with a thickness of 800 or 1500 nm sandwiched between 30-nm-thick p-type and n-type µc-Si:H layers. 

All of the materials were thoroughly investigated in previous works [29,30,31]. For the present devices, the intrinsic absorber layer was deposited via VHF-PECVD at 100 MHz with hydrogen dilution H_2_/SiH_4_ = 12.5, power density of 140 mW and pressure of 0.5 Torr. The p-type µc-Si:H window layer was deposited by VHF-PECVD at 40 MHz with H_2_/SiH_4_ = 125, doping ratio TMB/SiH_4_ = 0.6%, power density of 100 mW and pressure of 1 Torr. The final n-type µc-Si:H layer was grown by standard PECVD at 13.56 MHz with H_2_/SiH_4_ = 170, doping ratio PH_3_/SiH_4_= 0.8%, power density of 240 mW and pressure of 2.5 Torr. The substrate temperature was fixed at 150 °C for all of the layers. The devices were completed with a ZnO:Al (80 nm)/Ag back reflecting contact deposited through a shadow mask, thereby defining the cell area (1 cm^2^).

### 5.2. Electrical Performance Characterization of the Cells

The devices were characterized by measuring the current density–voltage *J*(V) characteristics under illumination and the external quantum efficiency (EQE). The *J*(V) characteristics, from which the open-circuit voltage *V*_OC_ and the fill factor FF were determined, were measured using a dual-lamp WACOM solar simulator under standard test conditions (25 1C, AM1.5 g, 1000 W/m^2^). The EQEs were collected with a commercial specialized setup (Bentham PVE300, Bentham, Reading, UK) that allows measurement of the absolute spectral response through the use of pre-calibration with a supplied reference cell. For the measurement, a probe light with a spot size that is substantially smaller than the cell area is used. Because the devices are not electrically isolated from the surrounding, the EQE also enables a more precise determination of the short-circuit current density (*J*_SC_) with respect to *J*(V), where the contribution from lateral collection is non-negligible. Therefore, *J*_SC_ has been calculated from the EQE curves by convolution with the photon flux of the global air mass 1.5 (AM1.5 g) solar spectrum.

### 5.3. Morphological Analysis of the Cells

For the morphological analysis, atomic force microscopy (AFM, Agilent Technologies, Santa Clara, CA, USA) was used. The model employed was the Agilent 5420 SPM/AFM. The AFM was positioned on an active stabilization table (Accurion NANO Series—Active Vibration Isolation, Accurion, Goettingen, Germany) providing vibration isolation during the acquisition of the images.

Measurements were made in tapping mode, and the utilized probe was a Nanosensors PPP-NCHR (Nanosensors, Neuchatel, Switzerland), with a resonant frequency of approximately 288 kHz. The images were measured for a surface area of 10 × 10 µm^2^ with a resolution of 512 pt. and a scan speed of 0.1 ln/sec in closed-loop mode. The raw data were then processed using the image processing software Agilent PicoImage 6.2 (Agilent Technologies), which enabled an elaboration of the raw data with measure extraction and analysis. In particular, leveling of the surface, noise reduction and zooming were performed. A cross-section profile analysis was performed with a special tool that automatically computed the mean depth for each hole and the width of the hole/peak considering as a limit a percentage of the height for each hole/peak.

In addition, a FEI Quanta 200 3D dual beam equipment (Fei, Hillsboro, TX, USA) was used for cross-sectioning the solar cells and evaluating the thickness of the deposited layers. The apparatus integrates a high focused ion beam (FIB), a scanning electron microscope (SEM), and a gas injection system for platinum deposition (GIS). FIB operates with gallium ions accelerated at 30keV. Ions strike the sample and sputter atoms from the surface, thus defining cross sections for internal analysis. Solar cells were mounted on the holder tilted to 52°. A sacrificial layer of platinum was firstly deposited by means of GIS on the surface to protect the first layer from ion beam irradiation during cross-sectioning. Just below the Pt strip, a regular cross section was made by using 50pA as gallium ion emission current. Subsequently, a cleaning step at lower emission current was applied to remove the re-deposited material on the cross section wall. In order to image the cleaned cross sections, the holder was tilted to 0°.

### 5.4. Optical Performance Characterization of the Cells

The experimental setup employed for the optical characterization of solar cells mainly consists of a UV-VIS light source (Avantes-Avalight-DHS, Avantes, Apeldoorn, The Netherlands), a fiber optic spectrometer (Avantes-AvaSpec-3648, Avantes, Apeldoorn, The Netherlands) and a proper fiber optic reflection probe (Avantes-FCR-7UV200, Avantes). This probe is based on a close-packing arrangement of optical fibers, which consists of six illumination fibers around one read fiber; light from the source is coupled to the illumination fiber bundle and carried to the probe end. The sample reflects the light back to the read fiber, which in turn is connected to the spectrometer. This particular arrangement enables more energy to be coupled from the light source, thus increasing the signal level and avoiding a spectral dependency on the probe-to-sample distance (as otherwise expected in the case of a single fiber used both to illuminate and read, described by the Fabry-Perot effect) [32].

The fiber optic reflection probe was kept perpendicular to the solar cells supporting the sample at a distance of 3 mm. The spectral characterization was performed through the determination of the intensity of the light reflected from the sample as a function of the wavelength λ according to the following expression:

(1) $$R(\text{λ})=\frac{R_{\text{sample}}(\text{λ})-R_{\text{dark}}(\text{λ})}{R_{\text{ref}}(\text{λ})-R_{\text{dark}}(\text{λ})}$$

where *R*(λ) is the normalized reflectance at a given wavelength λ; *R*_sample_ is the reflectance under sample illumination; *R*_dark_ is the ground noise of the instrument, assessed under dark conditions; and *R*_ref_ is the signal originating from an aluminum mirror used as a reference. The data acquired by the spectrometer were digitized, saved and plotted on a PC through a computer interface.

### 5.5. Numerical Simulations

In our numerical analysis, we used the commercial software package Comsol^©^ Multiphysics (RF module v. 3.5a), which is based on the finite element method [33].

The computational domain is limited by a quarter grating unit cell. By exploiting the crystal symmetry, one quarter of a cell was transversely terminated with two horizontal, perfectly electrically conducting and two vertical, perfectly magnetically conducting walls to simulate a normally incident plane wave with a vertically polarized electric field. The port conditions are applied at the top (air layer) and bottom (silver layer) boundaries.

The complex refractive index of Ag was taken from [34], and we modeled ZnO (1.96-0.002i @ 500 nm and 1.77-0.01i @ 950 nm) and µc-Si:H (4.179-0.2211i @ 500 nm and 3.388-0.0004i @ 950 nm) through spectroscopic ellipsometry on films deposited on glass.

The light absorption A was calculated through integration over the silicon volume of the energy loss considering an incident power of 1 W:

(2) A(λ)=∫VsiQlossdV=12∫VRe{(σE+jωD)E∗+jωB⋅H∗}dV

where *Q*_loss_ represents the energy losses and is equal to the sum of the resistive and magnetic losses.

Resistive heating *q* is defined as the scalar product of the current density *j* and the electric field vector *e*; therefore, the time-averaged resistive heating can be expressed as *Q*_loss_= <*q*> = <*j*·*e*>.

Specifically, using the time average theorem, we calculated the time-averaged resistive heating as a function of the current density and electric field in the phasor domain as

(3) Qav=12Re{J⋅E∗}

with * designating the complex conjugate. Then, from the constitutive equation of the current density (J=σE) and from the complex permittivity ε=ε′−jε′′=ε′−jσ/ω, we obtain the time-averaged resistive heating in the form

(4) Qav=12Re{σE⋅E∗}=12wε′′|E|2

where σ is the conductivity and ω is the angular frequency. 

The reflectivity has been calculated from the S-parameter as

(5) $$R=\text{abs}{\left\{S_{11}\right\}}^{2}$$

The short-circuit current density is evaluated by considering a conversion efficiency of 100% from the absorption as

(6) $$J_{sc}=\frac{q}{c\cdot h}\int A(\text{λ})\cdot S(\text{λ})\cdot \text{λ}\cdot d\text{λ}\;\left[{\text{A/m}}^{\text{2}}\right]$$

where *q* is the electron charge, *c* is the light speed in vacuum, *h* is the Planck’s constant, and *S*(λ) is the AM 1.5 solar spectrum. [35].

## 6. Conclusions

In conclusion, we have conducted a comparative study on numerical models used to predict the absorption enhancement in thin-film μc-Si:H solar cells due to the presence of metallic structured back contacts. The metallic backreflector is able to excite, at specific wavelengths, hybrid plasmonic-photonic resonances in the intrinsic layer of the cell, which are mainly responsible for the absorption enhancement. 

To evaluate the effectiveness of the numerical models considered in our analysis, they have been applied to a case study; µc-Si:H solar cells with Si thicknesses of 800 and 1500 nm have been fabricated using a top-down approach onto a 2D nanoimprinted grating.

Two numerical models have been implemented. In the first simplified model, we have assumed uniform and exact reproduction of the grating at the different interfaces, whereas in the optimized model, the real shape of the texture and the morphological changes, occurring during cell growth, are considered. To this end, we have carried out an extensive morphological characterization (using AFM) on the fabricated cells, to determine the real shape of the structure after the deposition of the different layers. The results of the morphological analysis have thus been used to develop an optimized numerical model for predicting the solar cell performances. 

The fabricated cells have been both optically and electrically characterized, and the experimental results have been compared with the numerical predictions. 

We have found that the first model is unable to correctly predict the performance of the solar cells and is thus unsuitable for a proper design that attempts to obtain the optimized texture structure. The numerical prediction provided by the optimized model, instead, approaches the experimental data by capturing the broadband absorption enhancement as well as the absorptive resonance peaks.

Overall, our results demonstrate that the interaction between the texture at each interface in the multilayer structure plays an important role in the light trapping phenomenon, thereby strongly influencing the phase-matching conditions at the basis of the excitation of both photonic and plasmonic modes.
